# In vivo experience with natural scaffolds for myocardial infarction: the times they are a-changin’

**DOI:** 10.1186/s13287-015-0237-4

**Published:** 2015-12-06

**Authors:** Isaac Perea-Gil, Cristina Prat-Vidal, Antoni Bayes-Genis

**Affiliations:** ICREC (Heart Failure and Cardiac Regeneration) Research Lab, Health Sciences Research Institute Germans Trias i Pujol (IGTP). Cardiology Service, Hospital Universitari Germans Trias i Pujol, 08916 Badalona, Barcelona Spain; Department of Medicine, Autonomous University of Barcelona (UAB), Barcelona, Spain

## Abstract

Treating a myocardial infarction (MI), the most frequent cause of death worldwide, remains one of the most exciting medical challenges in the 21st century. Cardiac tissue engineering, a novel emerging treatment, involves the use of therapeutic cells supported by a scaffold for regenerating the infarcted area. It is essential to select the appropriate scaffold material; the ideal one should provide a suitable cellular microenvironment, mimic the native myocardium, and allow mechanical and electrical coupling with host tissues. Among available scaffold materials, natural scaffolds are preferable for achieving these purposes because they possess myocardial extracellular matrix properties and structures. Here, we review several natural scaffolds for applications in MI management, with a focus on pre-clinical studies and clinical trials performed to date. We also evaluate scaffolds combined with different cell types and proteins for their ability to promote improved heart function, contractility and neovascularization, and attenuate adverse ventricular remodeling. Although further refinement is necessary in the coming years, promising results indicate that natural scaffolds may be a valuable translational therapeutic option with clinical impact in MI repair.

## Introduction

Myocardial infarction (MI) occurs when coronary artery blood flow is blocked. Currently, MI remains the most frequent cause of death worldwide [1]. In the United States alone, approximately 8 million people per year have a MI episode [2]. For effective MI treatment, it is necessary to limit adverse ventricular remodeling, attenuate myocardial scar expansion, enhance cardiac function and regeneration, and preserve synchronous contractility. Among the current therapies, only heart transplantation can fully achieve all these outcomes. Nonetheless, transplantation is highly limited by heart donor availability and host immunological response against the donated organ [3].

An alternative, novel therapeutic option is to deliver cells into the injured myocardium; this approach was demonstrated to be safe and feasible [4, 5]. To date, several cell types have been used for cardiac regeneration, including embryonic stem cells (ESCs) [6], cardiomyocytes (CMs) derived from induced pluripotent stem cells (iPSCs) [7], mesenchymal stem cells (MSCs) [8], bone marrow MSCs [9], cardiac stem cells [10], cardiac progenitor cells [11], skeletal myoblasts [12], endothelial cells (ECs) [13], adipose tissue-derived stem cells (ATDSCs) [14], and CMs [15]. However, modest results have been obtained due to massive cell loss after administration, low cellular survival or lack of cellular effect triggered by hypoxic conditions in the host tissue, failure to establish electrical or mechanical heart coupling, which results in arrhythmias, and low rates of cell differentiation into a cardiac lineage [3]. To overcome these limitations, new methods for enhancing the final outcome have been proposed.

Cardiac tissue engineering offers a plausible solution to the drawbacks encountered previously. This alternative consists of seeding cells onto a structural, supportive platform, known as a scaffold, and may also be supplemented with cytokines, growth factors, or peptides. The scaffold provides a biomimetic environment which resembles the physiological cardiac environment; thus, it favors cell attachment and differentiation, and it avoids direct administration of cells into an adverse environmental niche (that is, infarcted myocardium) [16, 17]. Therefore, an optimal scaffold for cardiac repair should recreate the myocardial microenvironment, structure, and three-dimensional organization, permit vascularization to ensure oxygen and nutrient flow to the cells, match electrical and mechanical requirements for proper host tissue coupling, be easily replaceable, and enhance cell survival and engraftment [3, 16, 17].

Depending on the origin of scaffold material, scaffolds are divided into two groups: natural and synthetic. Although synthetic materials offer the ability to directly control and adjust scaffold properties, natural materials appear to be more biodegradable and biocompatible. In addition, natural materials can better recreate the native myocardial microenvironment [18], which is necessary for generating the optimal, most suitable scaffold.

Here, we review natural scaffolds and hydrogel applications developed to repair injured myocardium after a MI. We describe constructs of natural materials combined with different cell types and other elements, and we analyze the main outcomes of heart function recovery in pre-clinical MI models and in clinical trials currently available (Tables 1, 2, 3, 4, 5 and 6). This summary provides an in-depth view of the current state of natural scaffold use in cardiac tissue engineering. Finally, we discuss the positive and negative aspects of the latest investigations in the field of myocardial regeneration.

**Table 1:** The principal in vivo studies using a collagen-based scaffold and the outcomes obtained

Scaffold material	Cell lines and/or other components	MI model	Main results	References
Collagen	–	Mouse	Negative ventricular remodeling prevented, deterioration of heart function prevented, lack of inflammatory response, angiogenesis↑, fibrosis↓, cell death↓	[26]
	–	Mouse	Patch attached, colonization of patch by native cells, EF↑, FS↑, LV internal diameter↓, LV posterior wall dimension↑, fibrosis↓, dilatation of LV chamber↓, angiogenesis↑, no immunological response	[27]
	–	Rat	LV dilatation↓, LV inner and outer diameters↓, LV pressure-volume curve shift (to the left towards control), angiogenesis↑	[28]
	Rat MSCs+interleukin-10	Rat	LV EF↑, apoptosis↓, infarcted wall average thickness↑, ratio collagen III/I↑, regulatory macrophage markers↑	[29]
	Rat ATDSCs	Rat	Evaluation of inflammatory response to diverse collagen scaffolds (non-crosslinked or crosslinked), presence of cells in the non-crosslinked scaffold	[30]
	Rat ATDSCs	Rat and pig	Rat: cell engraftment↑, LV EF↑, stiffer mechanical behavior, fibrosis↓, revascularization↑. Pig: LV EF↑, fibrosis↓, vascularization↑	[31]
	Sheep adipose tissue MSCs	Sheep	LV end-diastolic dimension improvement, diastolic function↑, angiogenesis↑, fibrosis extension↓	[32]
	Rat bone marrow MSCs	Rat	LV wall thickness↑, EF preservation, FS↑, fractional area change↑	[33]
	Rat bone marrow MSCs	Rat	Infarcted segment perfusion↑, infarct area↓, contractility↑, low inflammation, angiogenesis↑, ventricular wall thickness↑, LV dilatation↓	[34]
	Rat bone marrow MSCs+glycosaminoglycans	Rat	No inflammation, neovascularization↑, presence of cells	[35]
	Mouse Sca-1^+^ cells (collagen conjugated with anti-Sca-1 antibody)	Mouse	Number of infiltrated cells↑, capillary density↑, cell density↑, myocardium regeneration↑	[36]
	Human mononuclear bone marrow stem cells	Human	No mortality or related adverse effects, New York Heart Association functional class↑, LV end-diastolic volume↓, LV filling deceleration time improvement, scar area thickness↑, EF↑	[37, 38]
Collagen+chitosan	Encapsulated thymosin β4	Rat	Cardiac tissue loss↓, vascularization↑	[25]
	Integrin-binding, angiopoietin-1-derived peptide QHREDGS	Rat	Cardiac function↑, scar thickness and scar area fraction improved, presence of CMs↑, no inflammation	[39]
Collagen+oligo (acryloyl carbonate)- poly(ethylene glycol)-oligo(acryloyl carbonate)	Rat bone marrow MSCs	Rat	Preserved EF, infarct size↓, LV wall thickness↑, vessel density↑	[41]

*ATDSC* adipose tissue-derived stem cell, *CM* cardiomyocyte, *EF* ejection fraction, *FS* fractional shortening, *LV* left ventricle/left ventricular, *MI* myocardial infarction, *MSC* mesenchymal stem cell, *Sca* stem cell antigen

**Table 2:** Main achievements in myocardial infarction recovery after the administration of a fibrin scaffold

Scaffold material	Cell lines and/or other components	MI model	Main results	References
Fibrin	Rat skeletal myoblasts	Rat	FS and infarct wall thickness preservation	[52]
	Rat skeletal myoblasts	Rat	Infarct scar size↓, arteriole density↑	[53]
	Human cardiac and subcutaneous ATDPCs	Mouse	CM and EC differentiation, vessel density↑, LV EF↑, infarct size↓	[55]
	Rat bone marrow cells	Rat	Cell retention↑, LV perimeter↓, stroke volume and contractility preservation, cardiac function↑	[56]
	hESCs	Mouse and pig	Cardiac function↑, cell engraftment↑, angiogenesis↑, EF↑, infarct size↓, LV hypertrophy↓, systolic LV wall stress↓, LV systolic thickening fraction↓	[57]
	Rat adipose-derived MSCs	Rat	Preserved wall thickness, LV end-diastolic and systolic dimensions↓, LV end-diastolic volume↓, LV end-systolic volume↓, LV remodeling suppressed	[58]
	hESC-derived cardiac progenitors	Rat	LV end-systolic volume↓, EF↑, angiogenesis↑, absence of teratomas	[59]
	Human iPSC-derived CMs, ECs, and smooth muscle cells	Pig	Cell survival↑, LV EF↑, contractility↑, infarct size↓, regional wall stress↓, energetic efficiency↑, lack of arrhythmias, apoptosis↓, cellular expression of Nkx2.5↑, angiogenesis↑, immune response delayed, protective paracrine effects	[54]
	Rat heart cells; native population or CM-depleted population	Rat	EF and FS↑ (only for patch with native cell population), wall thickness↑, infarct size↓, cell migration, vascularization↑, electrical coupling and alignment not achieved	[60]
	Human umbilical cord blood MSCs	Mouse	Infarct size↓, vessel density↑	[61]
	Human umbilical cord blood MSCs	Mouse	Microvasculature formation↑, FS↑, EF↑	[62]
	Swine MSCs	Pig	LV thickness fraction↑, neovascularization↑, differentiation into myocyte-like cell lineage	[63]
	Swine MSCs+thymosin β4	Rat	Proliferation↑, protection against hypoxia, LV EF↑, LV FS↑, wall thickening↑, vasculogenesis↑, cell survival↑	[64]
Fibrin+PEG	SDF-1α	Mouse	c-kit^+^ cell recruitment↑, LV function↑	[68]
Fibrin+decellularized myocardial ECM	Human mesenchymal progenitor cells (TGF-β-conditioned or not)	Rat	Angiogenesis↑, cell migration↑, LV diameter and area preservation, contractility↑	[70]

*ATDPC* adipose tissue-derived progenitor cell, *CM* cardiomyocyte, *EC* endothelial cell, *ECM* extracellular matrix, *EF* ejection fraction, *FS* fractional shortening, *hESC* human embryonic stem cell, *iPSC* induced pluripotent stem cell, *LV* left ventricle/left ventricular, *MI* myocardial infarction, *MSC* mesenchymal stem cell, *PEG* polyethylene glycol, *SDF* stromal cell-derived factor, *TGF* transforming growth factor

**Table 3:** In vivo improvements achieved with scaffolds composed of the polysaccharides chitosan, alginate or hyaluronic acid

Scaffold material	Cell lines and/or other components	MI model	Main results	References
Chitosan	Rat brown ATDSCs	Rat	Cell survival and retention↑, EF↑, FS↑, LV end-diastolic pressure↓, LV pressure change↑, infarct size↓, fibrosis↓, ATDSC to cardiac lineage differentiation↑, vessel density↑, endothelial and smooth muscle cell differentiation	[81]
	Mouse ESCs	Rat	Infarct zone cell retention↑, ESC to cardiac differentiation, heart function↑, LV end-diastolic and end-systolic diameters↓, EF↑, FS↑, infarct size↓, wall thickness↑, complete chitosan degradation, microvessel density↑	[82]
	Mouse nuclear-transferred ESCs or fertilization-derived mouse ESC	Rat	For both cell types: infarcted area covered↑, possible differentiation into CMs, smooth muscle cells and ECs, heart function↑, LV end-diastolic and end-systolic diameters↓, EF↑, FS↑, infarct size↓, wall thickness↑, complete chitosan degradation, neovascularization↑	[83]
	bFGF	Rat	LV EF↑, LV FS↑, arteriole density↑, infarct size↓, fibrosis area↓	[84]
	RoY peptide	Rat	Angiogenesis↑, ventricular wall thickness↑, fibrosis↓, infarct size↓, LV FS↑, LV EF↑	[85]
Chitosan+alginate	Rat MSCs	Rat	EF↑, LV function↑, angiogenesis↑	[77]
	–	Rat	Angiogenesis↑, no inflammation exacerbation, apoptosis↓, presence of c-kit^+^ cells↑, proliferation↑, wall thickness↑, LV expansion↓, LV EF↑	[87]
Alginate	–	Rat	Absence of arrhythmias or thrombus formation, scaffold degraded, scar thickness↑, diastolic and systolic anterior wall thicknesses↑, LV end-diastolic and systolic dimensions↓, LV end-diastolic and systolic areas↓, cardiac dysfunction↓	[98]
	–	Dog	End-systolic and end-diastolic wall thicknesses↑, LV end-diastolic and systolic volumes↓, end-systolic sphericity index↑, LV EF↑, functional mitral regurgitation↓, LV function↑	[99]
	–	Pig	No arrhythmias or conduction blocks, no remote infarcts in other organs, LV enlargement↓, LV function↑, coronary blood flow not affected, scar thickness↑, anterior wall thickness↑	[100]
	Rat fetal cardiac cells	Rat	Vascularization↑, formation of myofibers and gap junctions, preservation of LV dimensions and FS	[102]
	Human ESCs or human embryonic bodies	Rat	FS↑, LV dilation, absence of inflammation, no cardiomyogenic differentiation, no cell retention	[103]
	RGD peptide	Rat	FS↑, LV dimension↓, LV wall thickness↑, angiogenesis↑	[105]
	RGD peptide+encapsulated MSCs (microbeads)	Rat	LV function↑, wall thickness preservation, LV internal dimensions preserved, infarct size↓, angiogenesis↑, high cell retention	[106]
	Unmodified alginate; RGD or YIGSR peptide-modified alginate; or RGE peptide-modified alginate	Rat	Unmodified-alginate: scar thickness↑, attenuated LV systolic and diastolic dilatations, LV FS↑, fractional area change↑, LV expansion index↓ (compared with all peptide-modified alginates)	[107]
	IGF/HGF (microbeads)	Rat	Scar thickness preservation, infarct expansion index↓, scar collagen accumulation↓, vascularization↑, apoptosis↓	[110]
	–	Human	New York Heart Association functional class↑, Kansas City Cardiomyopathy Questionnaire score↑	[111]
Alginate+fibrin	–	Pig	LV posterior wall thickness↑, infarct expansion↓, extractable collagen↓	[115]
Alginate+Matrigel+omentum	Neonatal rat cardiac cells with SDF-1, IGF-1 and VEGF	Rat	Mechanical and electrical coupling, relative scar thickness↑, angiogenesis↑, infarct expansion index↓, FS and fractional area change preserved, LV end-diastolic and systolic dimensions↓	[117]
Alginate+polypyrrole	–	Rat	No inflammation, angiogenesis↑, myofibroblast population↑	[118]
Hyaluronic acid	Alone or with VEGF	Rat	Ventricle thickness↑, infarct size↓, apoptosis↓, vascularization↑, heart function↑	[121]
	Rat BMMNCs	Rat	Apoptosis↓, inflammatory response↓, EF↑, ventricular dilatation↓, scar size↓, collagen content↓, angiogenesis↑, cell differentiation into ECs	[124]
	Pig BMMNCs	Pig	LV EF↑, interventricular septum thickness↑, LV end-diastolic pressure and volume↓, contractility↑, scar size and length↓, fibrosis↓, high cell retention, neovascularization↑	[125]
	Rat bone marrow MSCs (esterified hyaluronic acid)	Rat	Construct integration, vascularization↑, fibrosis↓	[126]
	Pig bone marrow MSCs (esterified hyaluronic acid)	Pig	Inflammation↓, fibrosis↓, degeneration of cardiac cells↓	[127]
	Hydroxyethyl methacrylate, SDF-1α, mouse bone marrow cells	Mouse	Cell homing in the myocardium↑	[128]
	rTIMP-3	Pig	LV end-diastolic dimension↓, LV EF↑, wall stress↓, infarct expansion↓, wall thickness↑, LV end-diastolic volume preserved, myofibroblast number↑, collagen content↑	[130]
	Gelin-S	Rat	LV EF↑, LV FS↑, neovascularization↑, collagen deposition↓	[131]
	Methacrylic anhydride	Sheep	Regional wall thickness↑, infarcted area↓ (only for highly stiff scaffold)	[132]
	Methacrylic anhydride or/and hydroxyethylmethacrylate	Sheep	Wall thickness↑, vascularization↑, inflammation↑, LV end-systolic volume↓ (only for highly stiff, stable scaffold)	[133]
Hyaluronic acid+gelatin	Human cardiosphere-derived cells	Mouse	Cardiac function↑, LV remodeling and abnormal heart morphology↓, viable tissue↑, wall thickness↑, cardiac and endothelial cellular differentiation, cellular engraftment↑, neovascularization↑, apoptosis↓	[134]
Hyaluronic acid+silk fibroin	Rat bone marrow MSCs	Rat	LV inner diameter↓, wall thickness↑, FS↑, inflammation↓, apoptosis↓, vascularization↑, α-MHC expression↑, paracrine factor secretion↑	[135]
Hyaluronic acid+chitosan+silk fibroin	–	Rat	LV inner diameter↓, wall thickness↑, LV FS↑, angiogenesis↑, paracrine factor expression↑	[136]
Hyaluronic acid+butyric and retinoic acids	Human placenta-derived MSCs	Pig	Scar size↓, infarct core zone↓, angiogenesis↑, fibrosis↓, end-systolic wall thickening and circumferential shortening↑, high homology with healthy myocardium	[137]

*ATDSC* adipose tissue-derived stem cell, *bFGF* basic fibroblast growth factor, *BMMNC* bone marrow mononuclear cell, *CM* cardiomyocyte, *EC* endothelial cell, *EF* ejection fraction, *ESC* embryonic stem cell, *FS* fractional shortening, *HGF* hepatocyte growth factor, *IGF* insulin growth factor, *LV* left ventricle/left ventricular, *MHC* myosin heavy chain, *MI* myocardial infarction, *MSC* mesenchymal stem cell, *rTIMP* recombinant tissue inhibitor of matrix metalloproteinases, *SDF* stromal cell-derived factor, *VEGF* vascular endothelial growth factor

**Table 4:** Outcomes in function recovery after myocardial infarction following gelatin and Matrigel scaffold delivery

Scaffold material	Cell lines and/or other components	MI model	Main results	References
Gelatin	Fetal rat ventricular cells	Rat	Scaffold adhered to tissue, presence of blood vessels, cell to cell linking and spontaneous contraction, no cardiac function improvements	[140]
	Erythropoietin	Rabbit	LV end-systolic and end-diastolic dimensions↓, LV EF↑, FS↑, ±d*P*/d*t*↑, erythrocyte number↑, hematocrit↑, infarct size↓, fibrosis↓, infarct border zone capillary density↑	[142]
	bFGF	Rat	FS↑, infarct size↓, infarcted/non-infarcted wall thickness ratio↑, LV expansion index↓, capillary and arteriolar density↑, CM apoptosis↓	[143]
	bFGF alone or with human bone marrow-derived MSCs or human cardiosphere-derived cells	Pig	bFGF alone: arterial vessels↑, myocardial perfusion↑, LV EF↑. With human cardiosphere-derived cells: LV EF↑, infarct volume↓, wall motion↑, differentiation to CM↑. With human bone marrow-derived MSCs: LV EF↑, infarct volume↓	[144]
	Human cardiac-derived stem cells+bFGF	Human	No adverse side effects↑, LV EF↑, infarct size↓, maximal aerobic exercise capacity↑	[145], NCT00981006
Matrigel	–	Rat	Capillary density↑	[147]
	–	Rat	LV EF↑, contractility↑, infarct wall thickness↑, angiogenesis↑, c-kit+and CD43+ stem cell myocardial homing↑	[148]
	Rat adipose-derived stromal cells	Rat	LV EF↑, LV akinesis↓, contractility↑, infarcted area size↓	[149]
	Mouse ESCs	Mouse	Connexin 43 expression, graft/infarct area↑, FS↑, LV wall thickness preservation	[150]
	Mouse ESCs	Rat	FS↑, myocardial wall thickness↑, LV dilatation prevention, connexin 43 and α-sarcomeric actin expression	[151]
	Human ESC-derived CMs with prosurvival cocktail	Rat	Cell engraftment↑, LV end-diastolic and systolic dimensions↓, FS↑, EF↑, infarcted area wall thickening↑	[152]
	Mouse bone marrow-derived MSCs	Mouse	No improvements in FS, EF, or LV diastolic end volume	[153]
Matrigel+collagen	Rat H9c2 cardiomyoblasts alone, with VEGF, or with bFGF	Rat	Three groups: cell survival↑, LV wall thickness↑, LV EF↑, FS↑. No significant additional improvements were observed with VEGF or bFGF	[154]
	Rat myoblasts	Rat	Inflammatory response↑, FS↑, LV end-systolic diameter↓, scaffold vascularized	[155]
	Rat cardiac myocytes	Rat	No improvements in cardiac function or LV wall thickness, sarcomere integrity, vascularized and innervated graft, contraction preserved, electrical and mechanical coupling requires further evaluation	[156]
	Rat neonatal ventricular CMs	Rat	CM sarcomeric structural integrity, FS↑, anterior wall thickness↑, LV end-systolic diameter↓	[157]
	Rat neonatal heart cells	Rat	Non-delayed electrical coupling, dilatation↓, systolic wall thickening↑, FS area↑	[158]

*bFGF* basic fibroblast growth factor, *CM* cardiomyocyte; d*P*/d*t* change in pressure over time, *EF* ejection fraction, *ESC* embryonic stem cell, *FS* fractional shortening, *LV* left ventricle/left ventricular, *MI* myocardial infarction, *MSC* mesenchymal stem cell, *VEGF* vascular endothelial growth factor

**Table 5:** Myocardial infarction animal models and the progress in infarction regeneration for decellularized extracellular matrix-based scaffolds

Scaffold material	Cell lines and/or other components	MI model	Main results	References
Decellularized myocardial ECM	–	Rat	LV EF↑, LV bulging↓, infarct LV wall thickness↑, infarct expansion index↓	[166]
	–	Rat	Viable myocardium islands inside infarcted zone↑, no arrhythmia induction, proliferative cell density (mainly lymphocytes)↑, EF preservation	[167]
	–	Rat and pig	Rat: ECM biodegradable and biocompatible with host myocardium, absence of embolization or ischemia. Pig: LV EF↑, LV end diastolic and systolic volumes↓, contractility↑, global wall motion score↑, proportion of endocardial muscle↑, fibrosis↓, presence of neovascularization, unaltered cardiac rhythm or blood chemistry	[168]
Decellularized pericardium ECM	Rat bone marrow MSCs	Rat	LV cavity enlargement prevented, LV FS↑, LV end diastolic and systolic pressures improved, no apoptosis, microvessel density↑, differentiation to smooth muscle cells or myofibroblasts, growth factor expression and cytokine release↑	[176]
	Rat bone marrow MSCs	Rat	LV FS↑, LV end diastolic and systolic pressure improvements, LV dilatation↓, absence of apoptosis, blood vessel density↑, differentiation into smooth muscle cells or myofibroblasts	[177]
	bFGF	Rat	bFGF retention↑, arteriole density↑, confirmation of vessel functionality	[178]
	HGF fragment	Rat	LV remodeling prevention, fractional area change↑, arteriole density↑	[179]
Decellularized pericardium ECM+RAD16-I peptidic hydrogel	Porcine mediastinal ATDPCs	Pig	Infarct size↓, vascularization↑	[174]
SIS	–	Mouse	LV end systolic area↓, contractility↑, infarct size↓, capillary formation↑	[180]
	Rabbit MSCs	Rabbit	LV dimensions improved, anterior wall thickness↑, contractility↑, LV relaxation↑, vascular density↑, no immunological response, cardiac troponin T and α-smooth muscle actin expression	[181]
	bFGF	Rat	EF↑, LV end systolic and diastolic volumes↓, contractility↑	[182]
UBM	–	Pig	Smooth muscle cells↑, myofibroblast recruitment, inflammation↓, thrombus extension↓	[162]
	–	Dog	Myocyte recruitment with normal morphology and organization, myocyte proliferation↑, regional stroke work↑, systolic contraction↑	[185]
	Human MSCs (spheroid or non-manipulated)	Dog	Regional stroke work↑, systolic area contraction↑, organized sarcomeric structure	[186]

*ATDPC* adipose tissue-derived progenitor cell, *bFGF* basic fibroblast growth factor, *ECM* extracellular matrix, *EF* ejection fraction, *FS* fractional shortening, *HGF* hepatocyte growth factor, *LV* left ventricle/left ventricular, *MI* myocardial infarction, *MSC* mesenchymal stem cell, *SIS* small intestine submucosa, *UBM* urinary bladder matrix

**Table 6:** Detailed data of clinical trials in progress or completed using different natural scaffolds

Scaffold material	Study name	Cell lines and/or other components	State	Follow-up	Main results/objectives	References/clinical trial identifier
Collagen	MAGNUM	Human mononuclear bone marrow stem cells	Completed with 20 patients	10 months	No adverse related events, 1 point reduction of New York Heart Association functional class, 26 % reduction of LV end-diastolic volume, 22 % improvement of LV filling deceleration time, 50 % increase of scar thickness, 26 % enhancement of EF	[37, 38]
Fibrin	ESCORT	hESC	Recruiting patients	–	Study the number and nature of adverse events (clinical/biological abnormalities, arrhythmias and cardiac or extracardiac tumors). Test feasibility and efficacy of the scaffold in cardiac function recovery	NCT02057900
Alginate	AUGMENT-HF	–	Completed with 6 patients	3 months	Increase of Kansas City Cardiomyopathy Questionnaire from 39.4 to 74, number of patients with New York Heart Association class III/IV reduced from 6 to 1. No improves in EF and LV end-diastolic and end-systolic volumes	[111]
Gelatin	ALCADIA	Human cardiac-derived stem cells+bFGF	Completed with 6 patients	6 months	12 % increase in LV EF, 3.3 % decrease of infarct size, maximal aerobic exercise capacity enhanced by 4.5 ml/kg/min	[145], NCT00981006
SIS	–	–	Enrolling participants (by invitation only)	–	Evaluate scaffold safety and beneficial effects in heart function	NCT02139189

*bFGF* basic fibroblast growth factor, *EF* ejection fraction, *hESC* human embryonic stem cell, *LV* left ventricle/left ventricular, *SIS* small intestine submucosa

## Natural scaffold materials for myocardial regeneration

**Fig. 1:**
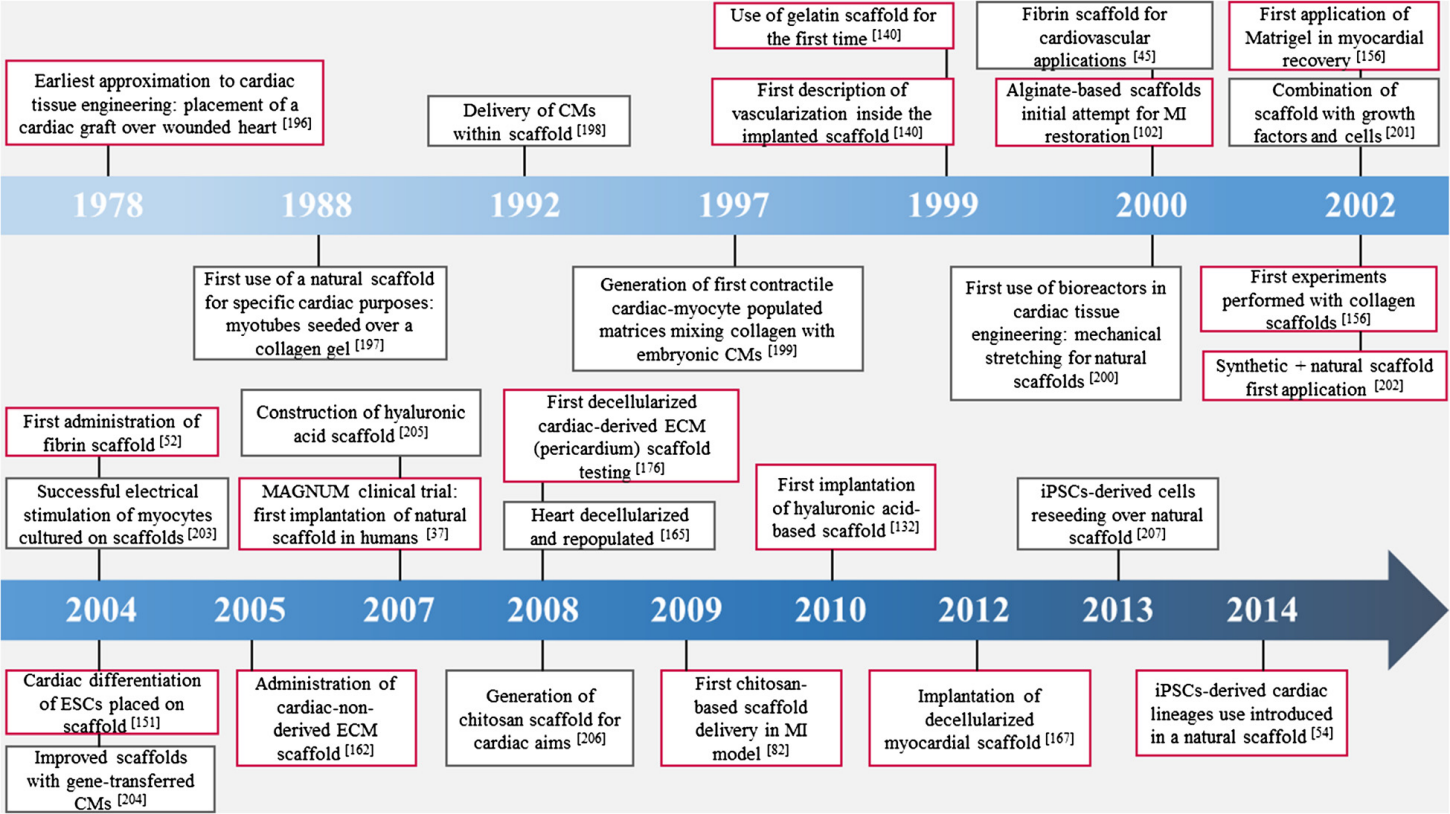
Milestones in the history of natural scaffolds in cardiac tissue engineering for myocardial infarction treatment. *Boxes with a grey outline* refer to natural scaffolds used in vitro. *Boxes with a red outline* indicate in vivo highlights related to natural scaffold application. *CM* cardiomyocyte, *ECM* extracellular matrix, *ESC* embryonic stem cell, *iPSC* induced pluripotent stem cell, *MI* myocardial infarction

**Fig. 2:**
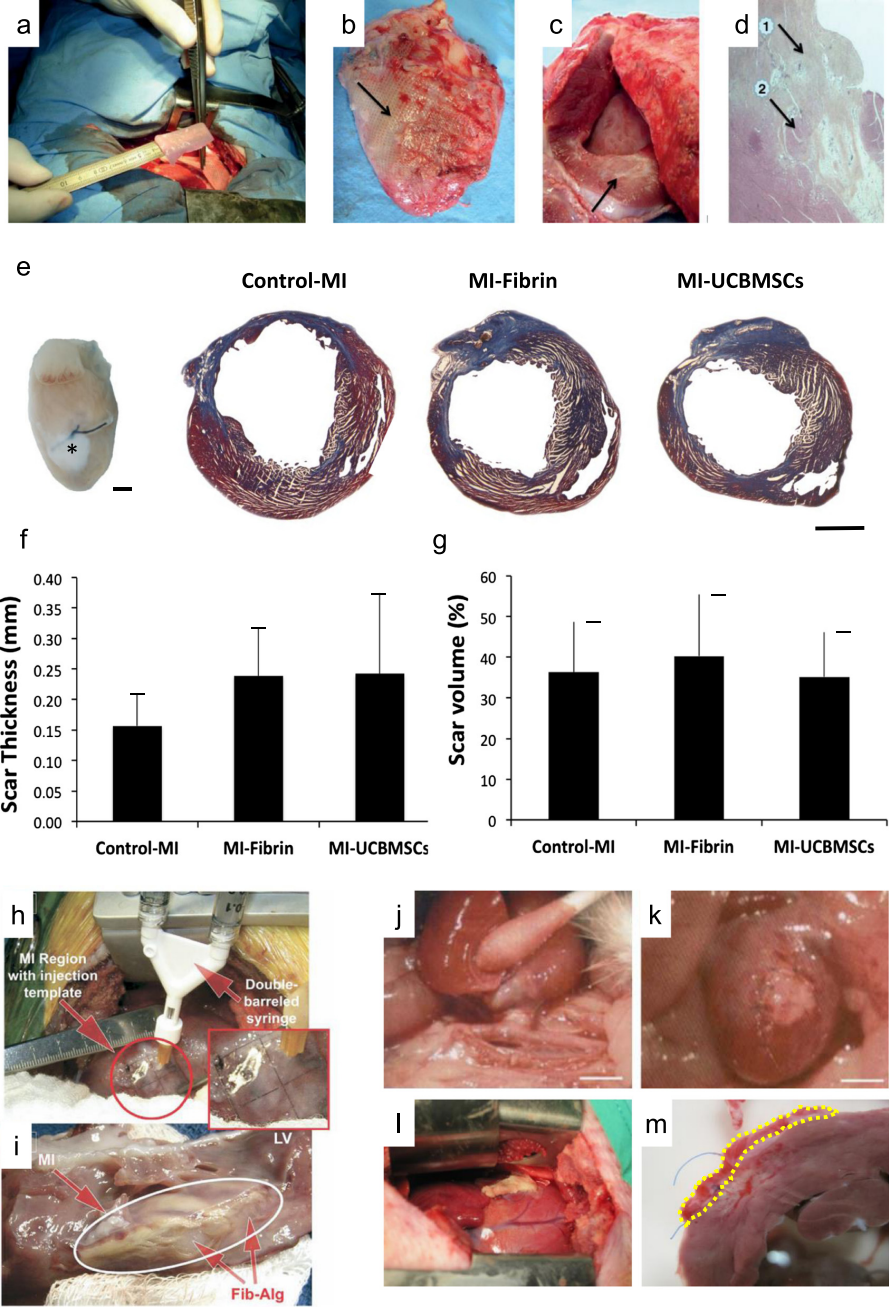
Natural scaffolds for cardiac tissue engineering. Combined surgical procedure using CorCap ventricular constraint device and collagen scaffold implantation in a sheep ischemic model for myocardial repair and ventricular chamber remodeling. **a** Introduction of the cell-seeded collagen matrix between the heart and the CorCap polyester device (Shafy et al*.* [32]). **b** Autopsy at 3 months showing the CorCap mesh covering both ventricles (*arrow*) (Shafy et al*.* [32]). **c** Left ventricular infarct scar (*arrow*) (Shafy et al*.* [32]). **d** Histology at 3 months of the ischemic/reperfused myocardium. *Arrows* show the mixed configuration: patchy fibrosis (1) and subnormal myocardium (2) (Shafy et al*.* [32]). **a**–**c** Reproduced, with permission, from [32]. **e**–**g** Three-dimensional engineered fibrin-cell patches implanted over infarcted myocardium wounds in mice. **e** Representative photograph of a mouse heart excised from a post-myocardial infarction (*MI*) animal at 4 weeks post-implantation of an adhesive fibrin-based patch composed of human umbilical cord blood mesenchymal stem cells (*UCBMSCs*) (*asterisk*). Images of Masson’s trichrome staining of cross-sections from the three groups of post-infarcted animals. Scale bar = 1 mm (Roura et al*.* [62]). Histograms represent the percentage of LV scar thickness (**f**) and volume (**g**) (Roura et al*.* [62]). **e**–**g** Reproduced, with permission, from [62]. **h** Intraoperative injection of the fibrin–alginate composite was performed using a 2 × 2 cm template with injection sites arrayed at 0.5 cm intervals within the region of MI. **i** At necropsy, the fibrin–alginate (Fib–Alg) could be visualized as amorphous densities within the MI region (*LV* left ventricle) (Mukherjee et al*.* [115]); reproduced, with permission, from [115]. **j**, **k** Heterotopic heart transplant surgery and hyaluronan-based scaffold (HYAFF^®^11) implantation in the rat MI model. The heart–lung block was carefully excised, the left lung removed, and the cardiac infarction induced by left descending coronary artery ligation on the bench (Fiumana et al*.* [126]); reproduced, with permission from [126]. **j** The allograft was transplanted by end-to-side anastomosis of the aorta to the abdominal aorta of the recipient. Scale bar = 5 mm (Fiumana et al*.* [126]). **k** The bioengineered HYAFF^®^11 was introduced into a pouch made in the thickness of the ventricular wall of the heterotopic heart at the level of the post-infarction scar. Scale bar = 5 mm (Fiumana et al*.* [126]). **l**, **m** Myocardial bioprosthesis implantation in porcine infarcted hearts; reproduced, with permission, from [174]. **l** A myocardial bioprosthesis, composed by decellularized human pericardium embedded with RAD16-I and mediastinal adipose tissue-derived progenitor cells, was implanted over the ischemic myocardium (Prat-Vidal et al*.* [174]). **m** Transversal heart section of a treated pig with the attached bioprosthesis indicated (*dotted yellow line*) (Prat-Vidal et al*.* [174])

In recent years, in vitro studies have consolidated our understanding of natural scaffold generation and their application in cardiac tissue engineering. This progress has enabled further investigation and improvements in this field, leading ultimately to in vivo progressive implantation of the developed scaffolds, supported by the positive results obtained in vitro (Fig. 1). In the following sections we review the most notable and latest improvements for in vivo MI treatment using different natural scaffold implantation methods (Fig. 2).

### Collagen

Collagen, the predominant protein in mammalian extracellular matrix (ECM), provides structural support for maintaining tissue integrity and contributes to the specificity of ECM microenvironments [19]. Several optimal properties, such as being biocompatible, adhesive, suturable, porous and readily combined with other materials, have made collagen appropriate for use as a natural scaffold in tissue engineering applications [20–25].

In cardiac tissue engineering, collagen scaffolds promote cardiac commitment, vascularization, and electrical coupling, thus representing a good candidate platform for MI repair. Collagen scaffold-associated benefits have been observed in different MI models (Table 1). Specifically, collagen type I delivery 3 hours after induction of a MI, without cells or added growth factors, can prevent adverse ventricular remodeling and long-term deterioration of heart function [26]. Furthermore, collagen can increase angiogenesis, reduce cell death, and limit the area of fibrosis, although these therapeutic effects were lost when the collagen scaffold was administered 1 or 2 weeks post-MI [26]. Another study revealed similar results when they inserted a collagen type I patch into rats subjected to MI [27]. After 4 weeks, compared with infarcted rats without the collagen patch, those with the patch had reduced adverse ventricular remodeling, limited fibrosis propagation, and significantly greater blood vessel formation. Remarkably, cardiac function was significantly increased, with an improvement of approximately 25 % in ejection fraction (EF) compared with non-treated infarcted animals [27]. These results were also confirmed in a rat MI model where the collagen type I-treated group showed attenuated left ventricular (LV) remodeling and increased angiogenesis, which promoted the formation of new connective vasculature between the scaffold and the host myocardium, pointing out the importance of the collagen scaffold itself [28].

In addition, constructs combining different growth factors, proteins, cells or other natural or biological materials onto collagen scaffolds have been placed over infarcted myocardium (Table 1). One study in rats delivered a collagen type I scaffold combined with MSCs and interleukin-10 to the infarcted area of the heart [29]. When analyzed 28 days after treatment, the group that received the scaffold combination exhibited higher EF (mean recovery of 7 %), a 40 % increase in infarcted wall thickness, a higher collagen III/I ratio, and less apoptosis inside the implant compared with the group that received the scaffold alone. Of interest, lower CD80+ macrophage and higher regulatory CD163+ macrophage infiltration was detected in the ischemic zone when the scaffold was applied, suggesting less associated inflammatory response. Nonetheless, these parameters were not significantly different when rats were treated with MSCs combined with the collagen scaffold but without interleukin-10, lacking the expected combinatory positive effect of cells plus interleukin-10 [29]. Another study compared a non-crosslinked collagen scaffold and crosslinked collagen scaffolds (both collagen type I) with variable degrees of crosslinking to modulate the material stiffness [30]. All were seeded with ATDSCs and sutured into rats with cardiac infarction. Only the non-crosslinked scaffold presented high biocompatibility and complete adhesion to the heart; a mild inflammatory reaction was observed 7 and 30 days after implantation. Moreover, this scaffold retained approximately 25 % of the seeded cells. However, heart contractility, cardiac function, and cell growth and survival were not assessed for all the scaffolds [30]. These gaps in assessment were partially covered in a later study which showed that, at 1 week and 1 month after implantation, the non-crosslinked collagen scaffold seeded with ATDSCs displayed more cell engraftment than ATDSC administration alone [31]. In addition, in both rat and pig chronic MI models, the groups that received both collagen and cells had significant increases in heart function (approximately 16 %) and revascularization, and a significant decrease in fibrotic area 4 months post-treatment compared with untreated animals and animals treated with either scaffold alone or cells alone. The reduction in collagen content of treated animals could be explained by the lower detected levels of procollagen C-proteinase and lysyl oxidase, reducing collagen crosslinking [31]. Similar results were obtained in a sheep MI model, in terms of cardiac function recovery and revascularization [32], thus indicating the suitability and promise of combined administration of collagen scaffold and ATDSCs. In addition, bone marrow MSCs combined with a collagen type I platform also preserved heart function after up to 6 weeks of treatment, in contrast to infarcted groups that were not treated or treated with cells only [33]. Further experiments demonstrated that bone marrow MSCs in a collagen type I scaffold had beneficial effects on contractility, wall thickness, angiogenesis, and infarcted area perfusion, and curbed ventricular dilatation and infarct zone expansion [34]. Moreover, when the collagen type I and bone marrow MSC combination was supplemented with glycosaminoglycans, the results demonstrated reduced inflammation, increased neovascularization, and improved retention of cells, but no LV function parameters were evaluated [35]. In another study, collagen type I was conjugated with antibodies that specifically recognized stem cell antigen-1 (Sca-1), a surface marker for hematopoietic, cardiac, and muscle stem cells. These antibodies enriched the scaffold by capturing Sca-1^+^ cells. When this scaffold combination was applied to the infarcted myocardium, it retained a high number of cells, increased cardiac tissue regeneration, and expanded capillary density compared with infarcted hearts that did not receive the enriched scaffold. It is important to emphasize that scaffolds with Sca-1 showed faster degradation of the collagen scaffold, and fiber arrangement was better organized [36].

In the Myocardial Assistance by Grafting a New bioartificial Upgraded Myocardium (MAGNUM) clinical trial, the delivery, safety, and effectiveness of a collagen type I scaffold loaded with autologous bone marrow MSCs was tested in humans (Tables 1 and 6) [37]. In this study, a total of 20 patients with myocardial ischemia that displayed indications for bypass surgery were included and divided into two treatment groups: one group (n = 10) was treated with cells only, and the other group (n = 10) received the collagen scaffold with cells. After completing a 10-month follow-up, no adverse events or death occurred. With both treatments, patients experienced improvements in EF and the New York Heart Association functional class. Compared with the group treated with cells alone, the scaffold-plus-cells treatment group showed enhanced LV end-diastolic volume, LV filling deceleration time, and scar area thickness, which indicated LV function improvement and limited adverse remodeling. Therefore, the collagen scaffold with cells was demonstrated to be both safe and effective for treating ischemia in humans compared with administration of cells alone [38]. These positive results should encourage further investigation following administration of combined collagen scaffold and MSCs, recruiting more patients and extending the clinical trial follow-up.

With regard to combinations of natural or biological materials, a mixture of chitosan, collagen type I, and encapsulated thymosin β4 was used to treat a rat MI model, with diverse results (Table 1). At 3 weeks after MI induction, treated rats showed reduced tissue loss (only 13 % compared with 58 % and 30 % for non-treated and thymosin β4-free hydrogel-treated animals, respectively) and enhanced vascularization compared with untreated animals or thymosin β4-treated animals, but no functional benefits were achieved [25]. When the integrin-binding, angiopoietin-1-derived peptide QHREDGS was attached to the same collagen and chitosan scaffold, infarcted animals displayed abundant CMs, no inflammatory response, and, more importantly, improved cardiac function [39].

Finally, collagen combined with synthetic materials would retain the properties of collagen (that is, degradability and compatibility) and could provide a means to recreate a natural, appropriate microenvironment, which could enhance proliferation, survival, and cardiac differentiation [40]. An injectable, hybrid hydrogel was created by combining collagen type I with the copolymer oligo(acryloyl carbonate)-poly(ethylene glycol)-oligo(acryloyl carbonate); cultured bone marrow MSCs were then added and the refilled scaffold was tested in a rat MI model (Table 1). Infarcted rats injected with this hybrid hydrogel plus MSCs exhibited an approximately 26 % reduction of the infarct area, a sixfold ventricular wall thickness enhancement, and increased vessel density compared with untreated infarcted rats. Notably, EF values reached those measured prior to MI induction, showing excellent heart function recovery for treated rats [41].

It is important to point out that all the in vivo studies which used collagen scaffolds were performed with type I collagen; thus, the observed differences between the different parameters can not be attributed to the collagen type. It would be interesting to carry out future animal experimentation using other collagen types (that is, collagen type III), evaluating the final outcomes and comparing them with the extensive work done with type I collagen scaffolds.

### Fibrin

Fibrin, a truncated form of fibrinogen, attracts and recruits leukocytes, principally macrophages, to participate in blood clot formation and wound healing processes [42–44], and also plays important roles in cell matrix interactions, inflammatory responses, and neoplasia [42].

Fibrin can be obtained from patient blood, which avoids the risk of adverse immunological responses, and can be easily manipulated by readjusting fibrinogen concentrations and/or polymerization rates to modulate matrix density, mechanical strength, and microstructure [45–49]. Moreover, fibrin scaffolds are good candidates for treating MIs due to their high biocompatibility, biodegradability, and capacity for incorporating different cell types. In addition, fibrin scaffolds can be assembled with either growth factors or other scaffold materials [49–51].

Due to its intrinsic properties, application of a fibrin patch alone (without cells) over the infarcted myocardium exerts beneficial effects (Table 2). In a rat MI model, application of a fibrin glue, which formed a scaffold, reduced the infarct size and increased microvessel formation. Similar results were observed when rat neonatal skeletal myoblasts were mixed with the fibrin glue. However, vessel density was greatest in the fibrin-alone group [52, 53]. Fibrin scaffolds have been most frequently used as a cell platform to test delivery of adipose-derived MSCs, bone marrow cells, ESC-derived cardiac progenitors, human iPSC-derived ECs, smooth muscle cells and CMs, a native cardiac cell population, umbilical cord blood MSCs, ESCs, and MSCs in different in vivo MI models (Table 2). Results have shown improved preservation of cardiac function post-MI, increased cell retention and, in some cases, reduced infarct size and enhanced angiogenesis [54–63]. The study by Ye and colleagues [54] used ECs, smooth muscle cells and CMs derived from iPSCs—the first in vivo use of iPSC-derived cells—and reported highly improved cardiac function (EF approximately 52 %) and contractility (thickening fractions of approximately 20 % and approximately 7 % at the border and infarct zone, respectively) compared with infarcted animals without treatment after 4 weeks [54]. Alternatively, fibrin scaffolds were enhanced by thymosin β4 encapsulation, which increased cell survival almost threefold, protected against hypoxic conditions, and improved cardiac function and wall thickness measured 28 days after MI [64].

Experiments in rat and non-human primate MI models, where a fibrin patch was applied with human ESC-derived cardiac progenitor cells, have produced convincing data that have led to approval of a first-in-human clinical trial (Tables 2 and 6) [65]. The study, entitled ‘Transplantation of human embryonic stem-cell derived progenitors in severe heart failure (ESCORT)’ (NCT02057900), is currently in phase 1 and recruiting participants. Its objective is to investigate the feasibility and safety of fibrin scaffolds combined with cells for treating patients with MI.

In order to increase the intrinsic low fibrin stiffness, fibrin scaffolds can be used in combination with other materials (Table 2) [47]. For example, when fibrin was mixed with the synthetic material poly(ether)urethane-polydimethylsiloxane or with poly (lactide-co-glycolide), in vitro experiments showed that it formed a suitable microenvironment which mimicked native myocardium, enhanced cell proliferation, and contributed to proper cell differentiation towards a cardiac lineage [66, 67]. Also, a hybrid polyethylene glycol/fibrin scaffold was combined with stromal cell-derived factor (SDF)-1α, a key factor in injured myocardium cell mobilization, and administered (without cells) into a mouse MI model [68]. This treatment promoted c-kit^+^ cell homing and increased EF and fractional shortening (FS), measured at 28 days post-MI. Nevertheless, no angiogenic activity was assessed and no significative reduction of infarct area was observed [68].

In another approach, fibrin scaffold combined with cardiac ECM provided acceptable cell viability, and its administration was feasible [69]. This scaffold was tested in vivo with mesenchymal progenitor cells injected into a nude rat MI model (Table 2). At 28 days after scaffold implantation in the infarcted myocardium, treated rats showed increased angiogenesis and cell migration, and preserved cardiac function compared with untreated animals. Next, the same scaffold was enhanced by preconditioning the MSCs with transforming growth factor (TGF)-β [70]. This treatment induced greater cell migration and vasculogenesis compared with MSCs not preconditioned with TGF-β, but no additional improvements in LV functionality were observed [70]. Therefore, combining fibrin with other materials could adjust the properties of fibrin itself and, to some extent, recreate the local stiffness, composition and fiber network present in the native myocardium, representing a good and plausible possibility for regenerating infarcted myocardium.

### Chitosan

Chitosan, a natural linear polymer obtained by chitin deacetylation, has been widely used for tissue replacement [71–76]. This natural material displays high biocompatibility and biodegradability and has the capacity to combine with conductive materials to improve electrical signal transmission and/or with other biomaterials [77, 78]. Additionally, chitosan was shown to be capable of high growth factor retention and strong cellular receptor adhesion due to its hydrophilicity [79, 80]; these properties make chitosan a suitable scaffold material for injured myocardial repair.

An in vivo study in a rat MI model also demonstrated that brown ATDSCs differentiated into cardiac lineage cells when applied into the infarcted area inside a chitosan scaffold, as they increased cardiac troponin I and T and connexin 43 expression (Table 3) [81]. This treatment resulted in improved cardiac function and contractility (better EF, FS and LV end-diastolic pressure), reduced infarct size and fibrotic area, and a remarkable increase in vessel density, measured 28 days after scaffold implantation [81]. Interestingly, ATDSCs partially contributed to this new vessel formation, showing von Willebrand factor- and α-smooth muscle actin-positive labeling. Altogether, chitosan plus ATDSCs appears to be a valuable approach for myocardial restoration.

Thermo-sensitive chitosan hydrogel (which polymerizes at body temperature) has been widely used with positive effects (Table 3). A rat MI model was treated with a thermo-sensitive chitosan hydrogel, combined with ESCs, nuclear transferred ESCs, or fertilization-derived ESCs, and exhibited enhanced heart function, increased vascularization in the damaged myocardium, and reduced infarct areas, measured 4 weeks after hydrogel transplantation. Importantly, implanted cells seemed to slightly differentiate towards CMs, smooth muscle cells and ECs [82, 83]. Temperature-sensitive chitosan was also applied, enriched with basic fibroblast growth factor (bFGF), in a rat MI model for 28 days [84]. The results included improved cardiac function, significant reductions in infarct size and fibrotic area (9.27 % and 22.91 %, respectively), and 2.7-fold more blood vessels in treated animals compared with controls. Another option is to modify the temperature-responsive chitosan scaffold by adding RoY peptide, a factor involved in cell proliferation, survival, and angiogenesis under hypoxic conditions. This construct exhibited satisfactory post-MI results, including infarct size reduction, angiogenesis promotion, ventricular wall thickness and cardiac function improvement [85].

When mixed with natural materials, chitosan scaffolds acquire other properties that favor cell maturation, adhesion, and scaffold coupling with the host myocardium (Table 3). Several natural materials have been tested with chitosan in cardiac tissue engineering, including myocardial ECM [86], alginate [77, 87], gelatin [88], collagen [89], and silk fibroin [90]. Chitosan mixed with alginate with MSCs was tested in vivo in a rat MI model, where it promoted improved cardiac function, new blood vessel formation, cellular survival, and cell proliferation [77]. Remarkably, the chitosan–alginate scaffold applied alone, without cell incorporation, provided the same beneficial effects in the damaged area after implantation. Of interest, the analysis of biodegradability displayed a low scaffold degradation rate, determined 8 weeks post-administration. However, an alginate-only scaffold provided better results in terms of wall thickness, LV expansion, and cardiac function than the chitosan–alginate scaffold, reducing the beneficial impact of the chitosan–alginate combination [87]. Moreover, despite the variety of possibilities and good results obtained with chitosan scaffolds, it is necessary to evaluate their effects in a large animal MI model to ensure that the described effects can be realized in a large cardiovascular system, similar to human. Therefore, further experimentation is mandatory to obtain satisfactory results combining chitosan with other materials.

### Alginate

Alginate is an anionic linear polysaccharide which can form a hydrogel through ionic crosslinking with divalent cations (mainly Ca^2+^) [91, 92]. This property also enables incorporation and retention of cells and proteins inside the hydrogel; thus, it can be used as a scaffold for tissue regeneration [93, 94]. Interestingly, the implantation of highly purified alginate, free of protein contaminants, resulted in a complete absence of adverse host immune response [95, 96]. Moreover, alginate mechanical behavior is easily modifiable by different crosslinking or by changing the molecular weight distribution to match the intrinsic stiffness of host myocardium [97].

Administration of alginate scaffolds alone resulted in significant improvements in cardiac function and increased scar thickness in various MI models, including rat [98], dog [99], and swine [100] (Table 3). Remarkably, alginate application was followed by the total absence of arrhythmias or thrombus formation [98, 100] and the replacement of the applied scaffold by connective tissue and myofibroblasts [98]. Pig models have enabled cell-filled scaffolds to be generated and tested in vivo with promising outcomes, including significative decreases in LV dilatation and LV mass, and a 53 % and 34 % increase in scar thickness and wall thickness, respectively [100]. Alginate scaffold hydrophilicity and porosity facilitate the incorporation and retention of cultured CMs on the scaffold (>90 % retention). These retained cells exhibited spontaneous contraction, which indicated that alginate platforms are suitable for cell seeding [101]. In a rat MI model, an alginate scaffold seeded with rat fetal cardiac cells enhanced neovascularization, preserved FS and end diastolic and systolic internal diameters, and promoted the formation of myofibers and cardiac gap junctions, measured 65 days after scaffold implantation [102]. On the other hand, not-so-positive results were obtained in vivo with human ESCs or embryonic bodies; neither new myocardium formation nor cardiomyogenic differentiation was observed in the implanted scaffold [103]. Additionally, treated animals developed LV dilatation and no ESCs were retained in the scarred area 3 weeks after injection. Nonetheless, the alginate composite did not trigger a deleterious immune response and FS increased by 4 % [103]. So far, fetal cardiac cells appear to be the most suitable cell source to continue investigation with alginate scaffolds.

Alginate scaffolds are typically modified, through integrin-mediated binding, with the addition of an arginine-glycine-aspartate sequence (the RGD peptide) derived from ECM proteins involved in cell adhesion, proliferation, migration, survival, and differentiation [104]. Promising results have been obtained in vivo (Table 3). A rat MI model showed remarkable enhancement of FS, LV dimension, angiogenesis, and LV wall thickness measured 5 weeks post-administration of modified scaffolds [105]. The RGD–alginate scaffold also promoted angiogenesis more effectively than the unmodified alginate scaffold in the animal MI model (12.6 ± 2.7 versus 9.3 ± 4.2 arteriole/mm^2^, respectively). Although not considered a scaffold, similar effects were described for RGD–alginate microbeads with encapsulated MSCs, evaluated 10 weeks after MI [106]. Nevertheless, in a comparative study, the unmodified scaffolds promoted better LV FS, greater fractional area changes, more attenuation of LV dilatation, a lower LV expansion index, and greater scar thickness increases compared with RGD-modified alginate scaffolds [107]. Thus, further experiments should be performed in vivo to elucidate under what conditions RGD has beneficial effects over unmodified alginate scaffolds and its effectiveness in improving cardiac function in order to determine the added value of RGD introduction.

For cardiac regeneration, alginate scaffolds have also been supplemented with two growth factors, insulin growth factor (IGF)-1, with cytoprotective effects, and hepatocyte growth factor (HGF), which is related to mainly anti-fibrotic and pro-angiogenic processes [108, 109]. When evaluated 4 weeks after MI, IGF/HGF plus alginate microbeads injected into the infarcted myocardium preserved scar thickness, reduced infarct expansion and fibrosis, enhanced angiogenesis and reduced cell apoptosis (Table 3) [110]. It would be interesting to test different combinations of growth factors with alginate scaffolds to determine whether they improve cardiac recovery post-infarction, and to explore the possibility of synergistic effects.

The ongoing AUGMENT-HF clinical trial (Tables 3 and 6), a first-in-human study, aims to evaluate the effects of alginate injection (Algisyl-LVR) in patients with dilated cardiomyopathy. An early follow-up at 3 months demonstrated the feasibility and safety of the scaffold injection. Cardiac evaluations demonstrated that patients who received treatment tended to show enhanced cardiac function and LV size, but no statistical differences were achieved compared with controls; in addition, treated patients showed significant improvements in quality of life and clinical status [111]. Future measurements at longer post-treatment times are expected to show significant beneficial effects on LV function parameters.

Alginate has been mixed with other biomaterials, including hyaluronic acid [112], gelatin [113], elastin [114], chitosan [77], fibrin [115], synthetic polymers [116], and omentum [117]. However, the effects of these combinations in pre-clinical MI models have not been fully defined in most cases (Table 3). One exception is the alginate plus Matrigel patch, assembled with neonatal rat cardiac cells and a growth factor supplement (IGF-1, vascular endothelial growth factor (VEGF), and SDF-1) [117]. This patch was pre-cultured in rat omentum for 1 week to induce pre-vascularization inside the patch prior to its engraftment into the infarcted area of a MI rat model. Then, 28 days after treatment with the pre-vascularized alginate–Matrigel scaffold, rats showed reduced LV dilatation, enhanced local angiogenesis, mechanical and electrical coupling with the host myocardium, limited LV dilatation, and improved cardiac function, preserving FS and diminishing LV dimensions [117]. In another study, a rat MI model was treated with polypyrrole added to alginate, then evaluated 5 weeks after the MI [118]. These rats exhibited increased angiogenesis and enhanced myofibroblast population recruitment compared with a control group treated with phosphate-buffered saline, and the presence of polymer was confirmed in the infarcted area with a non-associated inflammatory response. However, no functional benefits were assessed, and infarct size remained invariable after treatment, thus limiting its clinical application [118].

### Hyaluronic acid

Hyaluronic acid, a glycosaminoglycan component of the ECM, plays key roles in cell behavior and attachment, wound healing, inflammatory responses, tumor development, and connective tissue joining [119]. In addition to applications in damaged myocardium, hyaluronic acid-based scaffolds have been successfully used for space filling and wound repair, bone and cartilage restoration, nerve and brain regeneration, cell and protein delivery, and soft tissue and smooth muscle repair [120].

The molecular weight of the hyaluronic acid construct highly impacts infarcted myocardium recovery and its beneficial effects on cardiac function because the unit size affects mechanical properties, angiogenic processess, and other effects of the biomaterial itself [121, 122] (Table 3). A comparative study of scaffolds that comprised 50 kDa, 130 kDa, and 170 kDa hyaluronic acid units demonstrated that scaffolds composed of 50 kDa units showed the best effects, reducing apoptosis and infarct size from 29.4 % to 3.72 % and increasing ventricular wall thickness fourfold and heart function (LV end-diastolic pressure and Tau-weiss parameter), as analyzed 28 days after therapy [121]. These results were consistent with previous studies that described lower apoptotic rates and higher angiogenic activities with scaffolds composed of low molecular weight hyaluronic acid [122, 123]. Additionally, this study also evaluated the effects of the 50 kDa hyaluronic acid scaffold, alone or loaded with VEGF, on cardiac regeneration in both sub-acute and chronic MI animal models [121]. The effects on myocardial recovery and angiogenesis were similar between groups; therefore, VEGF addition did not act synergistically to achieve a significantly different outcome and hyaluronic acid is responsible for the described cardiac benefits [121].

In some cases, hyaluronic acid scaffolds have been seeded with cells of different lineages, mainly pluripotent stem cells, committed to differentiating into endothelial or cardiomyogenic phenotypes, which would reinforce the proangiogenic and regenerative impact of the scaffold. Taking advantage of hyaluronic acid scaffolds, which promoted high adhesion and proliferation, low apoptosis, paracrine factor gene expression, and regulated cell differentiation [124], experiments were conducted to assess the effectiveness of bone marrow mononuclear cells (BMMNCs) [124, 125] and bone marrow MSCs [126] for treating MI (Table 3). The combined action of the scaffold seeded with rat BMMNCs was tested in a rat MI model [124]. After 28 days, rats that received the scaffold plus BMMNCs exhibited less apoptosis, improved EF and LV internal dimensions, indicators of cardiac function, reduced macrophage and neutrophil infiltration and scar size, and enhanced angiogenesis compared with untreated rats. Interestingly, the BMMNC-seeded hyaluronic acid scaffold also induced better CM survival and cardiac output and smaller scars than an injection of BMMNCs alone or the hyaluronic acid scaffold alone. Furthermore, the hyaluronic acid scaffold promoted BMMNC differentiation towards ECs [124]. These outcomes were confirmed with porcine BMMNCs seeded in hyaluronic acid scaffolds and implanted into infarcted pigs; wall thickness, EF (increased 3.3 %), LV pressures and volumes and angiogenesis were improved while fibrotic area was reduced compared with untreated animals and animals treated with BMMNCs alone or hyaluronic acid scaffolds alone [125]. Alternatively, when harvested in an esterified hyaluronic acid scaffold, rat bone marrow MSCs promoted an increase in angiogenesis and a reduction in fibrosis in a rat MI model [126]. MSCs of porcine origin were also tested in swine [127]. In that study, the scaffold plus cell treatment induced a lower CD3 inflammatory response, less fibrosis by reducing the total amount of collagen I and III, and less cardiac cell degeneration compared with no treatment or treatment with a similar level of scaffold alone (except for inflammation attenuation) [127]. When bone marrow cells and SDF-1α were used to fill a methacrylated hyaluronic acid scaffold, bone marrow cell homing into the myocardium was increased approximately 8.5-fold, a higher value than that exhibited with administration of cells alone [128]. Nevertheless, despite all the data collected for bone marrow MSCs with hyaluronic acid scaffolds, function and other cardiac function parameters were not evaluated. Hence, the favorable results obtained with bone marrow MSC in hyaluronic acid scaffolds need to be corroborated in other studies to confirm these promising outcomes.

Other factors and compounds have been combined with hyaluronic acid scaffolds (Table 3). In one study, a recombinant tissue inhibitor of matrix metalloproteinases (rTIMP), which leads to adverse cardiac remodeling and fibrosis when deleted [129], was attached to the acid hyaluronic scaffold [130]. In infarcted pigs, the rTIMP plus scaffold treatment resulted in higher EF and wall thickness, less ventricular dilatation, reduced remodeling due to metalloproteinase activity, and a 50 % smaller infarcted area compared with untreated animals and compared with animals treated with the scaffold alone [130]. Thus, the addition of rTIMP seems to have had a positive and extra effect on cardiac regeneration post-MI. In another study, Gelin-S, a compound that enhances cell adhesion, was attached to the hyaluronic acid scaffold [131]. When applied to infarcted rats, this construct increased EF by 18.2 %, FS by 12.3 %, and neovascularization and decreased collagen deposition by approximately 50 %; however, this treatment was not compared with hyaluronic acid scaffold alone [131]. Finally, methacrylated hyaluronic acid scaffolds with different biomaterial stiffness (7.7 and 43 kPa) were injected into the infarcted myocardium to evaluate the impact of different mechanical properties on cardiac function and myocardial regeneration. Compared with controls, only the 43 kPa scaffold increased ventricular wall thickness and significantly decreased the infarct area (by approximately 20 %), although cardiac output and EF remained unchanged [132]. A similar study investigated the same hyaluronic acid scaffolds (approximately 7 kPa and 35 to 40 kPa) but in the context of low or high scaffold sensitivity to hydrolytic degradation [133]. Eight weeks after treatment, all scaffolds increased vascularization and inflammatory responses, but the ventricular wall thickened with more stable scaffolds, while LV systolic volume decreased with higher stiffness scaffolds. Thus, the results suggested that prolonged material stabilization provided the best benefits for myocardial restoration. The finding that optimal results were achieved with stiff scaffolds emphasized the notion that the mechanical properties of scaffolds play an important role in cardiac regeneration [133].

For cardiac repair, several supplementary materials have been combined with hyaluronic acid to improve scaffold properties, including gelatin [134], silk fibroin [135], chitosan plus silk fibroin [136], and butyric acid plus retinoic acid [137] (Table 3). A gelatin plus hyaluronic acid scaffold seeded with cardiosphere-derived cells was evaluated at 3 weeks after MI induction [134]. This construct induced increases in EF (approximately 17 %), viable tissue, wall thickness, and angiogenesis, enhanced cell survival, and promoted the uncompromised differentiation of cardiosphere-derived cells into endothelial and cardiac lineages compared with untreated animals and animals treated with cardiosphere-derived cells or scaffold alone [134]. Similarly, when silk fibroin plus hyaluronic acid scaffolds populated with bone marrow MSCs were injected into animal MI models, they reduced the LV inner diameter and inflammatory responses, and enhanced FS compared with animals with MI and without treatment [135]. Compared with untreated animals or animals treated with bone marrow MSCs alone, animals receiving the scaffold plus cells also exhibited increases in LV wall thickness, cell survival, alpha myosin heavy chain expression (a cardiac contractility protein), and release of VEGF, bFGF, and HGF paracrine factors. These results indicated that combining scaffold and cells produced synergistic effects [135]. In another study, a rat MI model was treated with chitosan plus silk fibroin plus hyaluronic acid scaffolds without cells [136]. At 8 weeks post-treatment, rats showed improved heart function parameters, increased angiogenesis, and upregulated expression of VEGF, bFGF, and HGF paracrine factors. These results suggested that the scaffold without cell seeding had positive effects on cardiac function, being a suitable scaffold for different cell harvesting and for determining scaffold effects independently of cellular ones [136]. Finally, infarcted pigs were treated with a combination of human placenta-derived MSCs placed in a hyaluronic acid scaffold that had been modified with butyric and retinoic acids [137]. This treatment resulted in increased FS, wall thickness, and blood vessel density compared with phosphate-buffered saline or cell-only treatment. Importantly, the collagen content was reduced after treatment, and scar size and fibrotic area core were reduced by 64 % and 44.6 %, respectively. Of interest, this study performed a proteomic analysis of the LV border zone; the group with the implanted scaffold displayed higher proteomic homology (45 %) to the healthy myocardium compared with the other groups, which corroborated the regenerative effects and functional recovery provided by scaffold delivery [137].

### Gelatin

Gelatin is a natural polymer that can be produced from bone, skin, or tendon collagen by partial hydrolysis with acid or alkaline solutions. Gelatin is highly biocompatible and biodegradable, has low antigenicity and can be produced and prepared at relatively low cost [138, 139]. These properties make gelatin ideal for use as a natural scaffold in cardiac tissue engineering.

Despite the small number of in vivo studies that used gelatin scaffolds alone or combined with other materials, some promising results are likely to encourage their future application (Table 4). For example, a gelatin mesh seeded with fetal rat ventricular cells was evaluated 5 weeks after implantation into infarcted rat hearts [140]. This construct showed good engraftment to the host myocardium, and the presence of blood vessels indicated vascularization of the cardiac graft. Furthermore, the cells rearranged to form connections between them, and exhibited spontaneous contractions. However, lack of functional or structural cardiac improvements dissuades further application of this particular combination [140]. The scaffold effects could be enhanced by adding erythropoietin, a glycoprotein hormone used in the treatment of anemia in patients with heart failure, which reduces ventricular hypertrophy and increases EF [141]. In a rabbit MI model, gelatin plus erythropoietin scaffolds were applied 20 minutes after inducing the MI [142]. At 14 days and 2 months after treatment, the treated rabbits displayed reduced LV diastolic and systolic dimensions, infarct size, and fibrosis, and enhanced EF, FS, and capillary density in the infarct border zone. In another study, bFGF was added to gelatin scaffolds for treating a rat MI model [143]. After 2 and 4 weeks of treatment, these animals showed lower CM apoptotic rates, higher arteriole densities, higher expansion indexes, greater infarcted/non-infarcted wall thickness ratios, and smaller infarct sizes in comparison with untreated animals (except for infarct size, which was only reduced after 2 weeks). Compared with scaffold alone, the scaffold plus bFGF treatment induced, at 2 weeks post-treatment, smaller infarct areas, lower expansion indexes, lower apoptotic rates, and higher arteriole numbers; at 4 weeks, more arterioles and capillaries and less apoptosis were observed [143]. In a pig MI model, bFGF was combined with the gelatin scaffold, with or without either human bone marrow-derived MSCs or human cardiosphere-derived cells [144]. When the three groups were compared (scaffold plus bFGF, scaffold plus bFGF plus human bone marrow-derived MSCs, and scaffold plus bFGF plus human cardiosphere-derived cells), optimal results were achieved with the combination of scaffold plus bFGF plus human cardiosphere-derived cells, which displayed the highest EF increase (approximately 9 %) and infarct volume reduction (approximately 3.7 %), the greatest wall motion index variation (approximately 13 %), and the most differentiation into CMs. Thus, the results confirmed that bFGF and the cells had additive effects [144].

Collectively, the outstanding results obtained with bFGF and MSCs in a gelatin scaffold have led to its implementation in humans. In a phase I clinical trial, ALCADIA (AutoLogous human CArdiac-Derived stem cell to treat Ischemic cArdiomyopathy, NCT00981006), patients with MI were treated with a combination of gelatin hydrogel embedded with human cardiac-derived stem cells and bFGF (Tables 4 and 6). Of six treated patients, one was excluded due to graft occlusion. Of the five remaining patients, only one experienced heart failure worsening. Evaluation after 6 months revealed a 12 % increase in the LV EF, an improvement in maximum aerobic exercise capacity, and a 3.3 % decrease in infarct size [145]. Nevertheless, a larger number of patients and a longer follow-up are required to evaluate treatment effectiveness.

### Matrigel

Matrigel is a biomaterial derived from ECM secreted by Engelbreth-Holm-Swarm mouse sarcoma cells [146]—whose composition has not been fully defined—which resembles and mimics myocardial ECM. Matrigel promoted angiogenesis both in vitro with ECs and in vivo [147]; thus, it appears to be a good candidate biomaterial for constructing a scaffold for cellular support.

Matrigel (without cells) was administered to infarcted myocardium to evaluate its effects on cardiac function and tissue regeneration (Table 4). In a comparative study, fibrin, collagen, or Matrigel was injected into a rat MI model [147]. Matrigel enhanced capillary density but did not improve cardiac function. On top of that, only collagen significantly increased myofibroblast infiltration compared with untreated animals [147]. Conversely, another study injected Matrigel alone into infarcted rats [148]. At 4 weeks after treatment, increases were observed in LV EF (improvement of 22.7 %), contractility (a 24.5 % enhancement of LV pressure decline), infarct wall thickness, angiogenesis, and recruitment of c-kit^+^ and CD43^+^ stem cells to the myocardium.

Taking advantage of the described regenerative effects of Matrigel, several studies have mixed Matrigel with different cell types (Table 4). In one study, Matrigel was combined with adipose-derived stromal cells and injected into a rat MI model [149]. At 4 weeks after the MI, treated rats showed increased normalized EF, less LV akinesis, increased heart contractility, and a smaller infarct area. ESCs have also been used extensively. In one study, a mixture of mouse ESC plus Matrigel scaffold improved FS, preserved LV wall thickness, and increased expression of the cardiac gap junction marker connexin 43 in comparison with untreated animals and animals treated with Matrigel alone [150]. Consistent with this study, a Matrigel plus mouse ESC combination was administered to infarcted rats and evaluated 2 weeks post-treatment [151]. Treated rats exhibited increased FS and LV wall thickness and reduced LV dilatation compared with untreated rats. In addition, introduced cells expressed the cardiac markers connexin 43 and α-sarcomeric actin. In spite of these results, a comparison between Matrigel alone and Matrigel with cells showed non-significant differences in terms of FS, LV wall thickness, and prevention of LV dilatation, outlining the rle of Matrigel in myocardial restoration [151]. Human ESC-derived CMs seeded onto a Matrigel scaffold and supplemented with a prosurvival cocktail were evaluated in a rat MI model [152]. Treated rats showed significantly enhanced cell engraftment in the scarred area compared with Matrigel plus cells alone. Additionally, at 4 weeks after treatment, the prosurvival cocktail complementation also revealed better results with regard to ventricular dilatation, FS, EF, and infarct zone wall thickness [152]. Conversely, when bone marrow-derived MSCs were mixed with Matrigel, only modest results were observed; there was no improvement in FS, EF, or other cardiac function parameters, hampering future research with these cells [153]. To sum up, extensive good results obtained with ESCs could lead next to in vivo testing in a porcine MI model, prior to clinical trials.

In most cases, Matrigel was combined with collagen, the primary structural protein of the ECM (Table 4). When these two compounds were combined with rat cardiomyoblasts, significant improvements were observed regarding cell survival, cardiac function, and LV wall thickness compared with controls and collagen-matrix treated animals [154]. When a growth factor, either VEGF or bFGF, was added to the Matrigel plus collagen scaffold, no other positive cardiac effects were obtained compared with Matrigel plus collagen alone [154]. Nevertheless, modest results were obtained in a similar study that also employed a Matrigel plus collagen cellular scaffold, but filled with myoblasts [155]. In this study, only a significant FS improvement was detected in infarcted rats after 4 weeks of treatment compared with pre-treatment (42 % versus 33 %); no significant effects were observed in other cardiac parameters. Moreover, the group that received the Matrigel plus collagen scaffold displayed a larger adverse immunological response than untreated rats or rats treated with fibrin [155]. In another study, cardiac myocytes were harvested and incorporated into a Matrigel plus collagen scaffold for treating a rat MI model [156]. This treatment did not provide any benefit in terms of heart function or LV wall thickness. Nonetheless, the generated scaffold was able to couple with the host myocardium, preserved contraction and sarcomere integrity, and presented high levels of neovascularization and innervation after engraftment. It is important to point out that although connexin 43 and cadherin expression were demonstrated, further investigation is necessary to show complete electrical and mechanical coupling [156]. The addition of CMs to a Matrigel plus collagen scaffold promoted FS and increased anterior wall thickness [157]. Also, at 4 weeks after MI induction, rats that received this treatment maintained sarcomere integrity and structure and exhibited decreased LV end-systolic volume in comparison with untreated infarcted rats or animals treated with either cells or scaffold alone [157]. Administration of neonatal rat heart cells supported by the Matrigel plus collagen scaffold also led to electrical assembly with the myocardium, diminished ventricular dilatation, enhanced ventricular wall thickening, and increased FS area [158]. Data collected suggest that a Matrigel–collagen scaffold combined with cardiomyoblasts or CMs should have a high impact in future investigations.

### Decellularized extracellular matrix

ECM consists of a dynamic blend of structural and functional molecules that are secreted by cells, with a slightly different composition depending on tissue source [159]. This physiological cellular support platform gives cells a suitable microenvironment; it guides cellular proliferation, attachment, differentiation, migration, and viability by providing different signals or cues [160, 161]. Hence, the isolation of an intact ECM would supply cellular support that best matched the native or physiological extracellular environment [18]. For ECM extraction, it is necessary to remove all cellular and nuclear content, in a process called decellularization, and the acellular ECM must maintain its integrity and architecture [3, 162]. Successful tissue decellularization requires the careful selection of physical, chemical, and enzymatic agents that can remove cellular material without disrupting the ECM. Decellularizing agents have variable effects on ECM structure and composition; any negative distortion of the matrix organization or integrity may affect its ability to support cells [163, 164]. Therefore, maximal cell removal and ECM property retention are mandatory for obtaining optimal ECM for use as a cellular scaffold. Among the essential properties to maintain, correct three-dimensional organization of the ECM helps in proper cell adhesion, differentiation, survival, and integration [18, 160, 164]. Closely related to this, mechanical properties are tightly associated with fiber arrangement and three-dimensional architecture, which in turn affects ECM scaffold coupling with the host myocardium and its synchronous contraction.

To date, many organs and tissues have been completely decellularized, including heart valves, myocardium, pericardium, lung, pancreas, kidney, liver, mammary gland, and nerve [163, 164]. For cardiac tissue engineering, myocardial ECM possesses the best properties; it can exactly recreate the microenvironment of the native myocardium. Thus, it favors coupling with host cardiac tissue when engrafted in the infarcted zone. The first heart decellularization was performed in 2008 by antegrade coronary perfusion with sodium dodecyl sulfate. The generated acellular organ preserved the primary matrix proteins, vascular architecture, valves, and chambers; when it was seeded with CMs and ECs, the recellularized heart exhibited contraction [165].

**Fig. 3:**
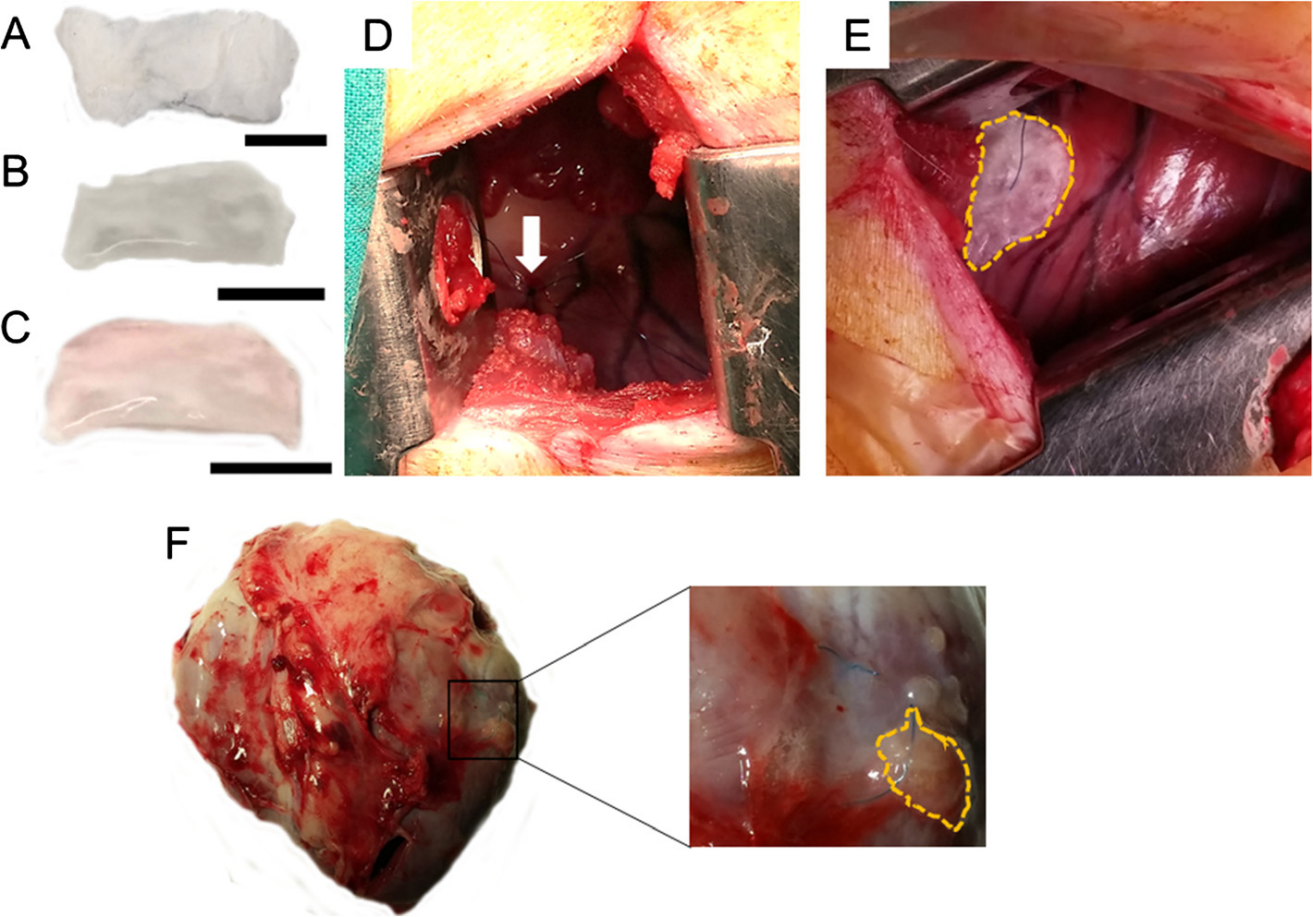
Engraftment of a decellularized myocardial ECM scaffold embedded with cells in a swine myocardial infarction model. **a** Lyophilized and gamma ultraviolet sterilized decellularized myocardial ECM scaffold. **b**, **c** Decellularized scaffold after the addition of peptide hydrogel (**b**) and porcine adipose tissue-derived progenitor cells (**c**). Scale bars = 1 cm. **d** Image of the myocardial infarction site, induced by double ligation in the first marginal branch of the circumflex artery (indicated with *white arrow*). **e** Reseeded decellularized scaffold placed over the injured myocardium. Scaffold is indicated with *yellow dotted lines*. **f** Presence of the implanted scaffold on the infarcted area in explanted hearts 28 days after sacrifice. The remaining scaffold is highlighted with *yellow dotted lines*

Several studies have been performed in vivo using decellularized myocardium ECM scaffolds (Table 5). Local administration of decellularized myocardial ECM was used to treat the infarcted area in a rat MI model [166]. Six weeks after ECM injection, heart function was enhanced through an 8 % elevation in LV EF, a 1.2-fold increase in LV wall thickness, and a 1.3-fold reduction in the infarct expansion index [166]. In another study, similar improvements were achieved with decellularized myocardial ECM application in a rat MI model; the EF was preserved, a higher proportion of viable myocardium was achieved (1.7-fold higher compared with controls), and arrhythmias were completely absent [167]. These results were corroborated when the decellularized myocardial matrix was administered to a porcine MI model [168]. In that case, EF and contractility were enhanced, LV volumes and fibrotic areas were reduced, and the proportion of endothelial muscle was increased. In addition, adverse side effects (ischemia, embolization, and altered heartbeat) were not detected in either pig or rat infarct models. These results ensured that scaffold delivery was a safe procedure; they also demonstrated that the ECM was highly degradable and biocompatible [168]. These promising results may open the door to repopulate decellularized myocardium with cells. A first attempt showed encouraging outcomes for future pre-clinical applications, even though they used a combination of decellularized myocardial ECM plus fibrin embedded with mesenchymal progenitor cells, and not the matrix alone plus cells [70]. Recently, our group has implanted a combination of decellularized porcine myocardial ECM (previously characterized and successfully recellularized in vitro) refilled with hydrogel and adipose tissue-derived progenitor cells (ATDPCs) in an infarcted porcine MI model [169] (Fig. 3). The scaffold remained in the damaged area 28 days after animals were sacrificed (Fig. 3f). Thus, this alternative scaffold is feasible and may provide new promising results using an acellular myocardial scaffold.

ECM derived from decellularized pericardium has also been widely used as a supportive scaffold for MI regeneration (Table 5). Pericardium ECM is a porous material that facilitates cellular retention and vascularization. It is easy to perform pericardium resection during surgery and subsequently extract the ECM; moreover, there are no negative consequences [170, 171]. Pericardium properties are comparable among human individuals, and these similar characteristics permit low variability among samples [172]. In vitro results have demonstrated that pericardial ECM seems to resemble myocardial ECM in structure and microenvironment, retain infiltrated cells, and support cardiac differentiation [170, 173–175].

In one in vivo study, rat infarcted myocardium was patched with decellularized pericardium and bone marrow MSCs and evaluated 12 weeks after treatment. The results revealed improvements in LV FS and LV pressures, infarct area vasculogenesis, cardioprotective growth factor and cytokine secretion, and cell differentiation into smooth muscle cells or myofibroblasts [176]. Nearly the same improvements were described when a decellularized pericardium sandwiched between multilayered sheets of bone marrow MSCs was applied to a rat MI model, highlighting functional enhancements in FS and LV pressures [177]. The three-dimensional organization apparently showed correct porosity and pore size, facilitating interconnectivity. Both studies confirm the suitability of MSCs with decellularized pericardial ECM, enabling us to move forward to large animal model experiments. Another study evaluated the synergistic effects of bFGF combined with decellularized pericardium, which displayed well-organized matrix fibers, for treating a rat MI model [178]. After injection, this combination resulted in higher bFGF retention and a 112 % higher number of functional blood vessels in treated rats compared with animals that did not receive bFGF and rats that received collagen alone, decellularized pericardium alone, bFGF alone, or bFGF combined with collagen. An increase in inflammation was observed, but this was attributed to bFGF, not to the decellularized pericardium, because injection of the latter alone did not show significant inflammation compared with saline-injected control groups [178]. When HGF was mixed with decellularized pericardium, treated rats showed significant prevention of LV remodeling, enhanced cardiac function (only fractional area change), and increased arteriole density [179]. Thus, it is imperative to continue investigating the use of growth factors to determine their effectiveness in heart function and structure recovery. Finally, when decellularized pericardium was assembled with RAD-16 peptidic hydrogel and ATDPCs in a swine MI model, infarct size was 36 % reduced and vascularization inside the scaffold was increased. Of note, the scaffold displayed non-disrupted fiber organization and a high in vitro biodegradability, losing about 70 % of the initial weight after 24 hours [174].

Other, unrelated cardiac ECMs have been used for cardiac restoration (Table 5). One of these is the acellular ECM derived from porcine small intestinal submucosa (SIS), which is highly biocompatible and mechanically modifiable. Injection of SIS alone in a mouse MI model provided good results: it maintained better LV geometry, reduced the infarcted area, and enhanced contractility and blood vessel density after treatment [180]. In a mouse MI model, treatment with SIS seeded with MSCs improved heart function (EF and LV diastolic and systolic dimensions), contractility, and angiogenesis, and attenuated wall thinning, LV enlargement and adverse immunological responses [181]. Notably, the SIS patch was partially degraded and the cells seeded on it seemed to differentiate into cardiac lineages because they expressed cardiac troponin T and α-smooth muscle actin; however, no connexin 43 expression was detected. This combination was more effective than SIS administration without cells [181]. When SIS was enhanced with bFGF, treated rats displayed higher EF (55.3 % versus 35.1 %), greater prevention of LV remodeling by reducing LV end-diastolic volumes, and improved heart contraction compared with the untreated group. Of interest, non-enhanced SIS only improved cardiac contractility compared with controls [182]. Currently, a clinical trial (NCT02139189) has begun to recruit participants to test the feasibility and safety of an SIS matrix (also called CorMatrix) for treating damaged myocardium (Tables 5 and 6).

Another non-cardiac-derived ECM widely used is the acellular urinary bladder matrix (UBM; Table 5). Extracted from urinary bladder, it is basically composed of type IV collagen, laminin, and entactin [183]. As a scaffold for cardiac repair, a UBM patch was used to repair an excised portion of the myocardium in a dog model [184]. This treatment enhanced ventricular function compared with a synthetic matrix patch composed of Dacron; additionally, CMs were detected in the UBM patch area, which was not observed with Dacron. In another study in a pig MI model, administration of a UBM patch without cells increased myofibroblast recruitment and the presence of smooth muscle cells, reduced the inflammatory response, and limited thrombus expansion compared with treatment with an expanded polytetrafluoroethylene synthetic biomaterial [162]. Moreover, UBM scaffold was not distinguished from the myocardium. Nevertheless, no improvements in cardiac function, LV dilatation, or contractility were achieved [162]. In a similar study performed in dogs, UBM enhanced regional stroke work and systolic contraction by 3.7 % and 4.4 %, respectively [185]. Myocyte recruitment and proliferation, measured 8 weeks after treatment, were also increased. Interestingly, myocyte arrangement in the UBM scaffold was similar to that in the neighboring myocardium [185]. Finally, a comparative study was conducted on UBM patches embedded with either spheroid-derived MSCs or unmanipulated MSCs in a dog MI model [186]. The results revealed that spheroid-derived cells provided better heart function and improved contractility, as assessed by regional stroke work, in comparison with unmanipulated MSCs. Myocytes present in the scaffold were correctly rearranged and showed an organized sarcomeric structure [186].

## Considerations and future perspectives

With all the collected data provided by the different studies, it is clear that engrafted scaffolds in the injured heart exert a beneficial effect over myocardium. In spite of not being the main point of this review, it is not fully understood how scaffolds promote the described regenerative effects and which mechanisms follow them. It has been described that a scaffold itself is capable of inducing new blood vessel and nerve fiber formation in swine myocardium, thus facilitating revascularization and reoxygenation of the hypoxic area and electrical coupling [187]. Probably, the local hypoxic environment in the infarcted area induces expression of vascular endothelial and platelet-derived growth factors, SDF-1α, and macrophage chemotactic protein, promoting recruitment of circulating cells to vascularize the affected myocardium in a more favorable microenvironment provided by the scaffold [187, 188]. The addition of cells helps to reinforce this effect by secreting paracrine factors and synthesizing new endogenous ECM, which in turns mobilizes more cells, favoring myocardial regeneration.

Hence, scaffold biodegradability is a key parameter, as scaffolds should not be degraded quickly after engraftment so they can continue to supply a suitable platform for initial cell administration. However, scaffolds should be easily degradable to allow them to be replaced by the new endogenous matrix synthesized by the administered cells. Moreover, components and molecules derived from degradation have to be non-toxic and absorbable by the body. In the specific case of the natural scaffolds reviewed here, elements derived from degradation are biocompatible and naturally absorbable. In most studies, however, the biodegradability parameter has not been determined. In the work reviewed here, only a few have shown detailed information about scaffold degradation rates, with non-exact quantifications and without clear criteria for determining the degradation rate of the scaffolds [36, 87, 168, 174, 181]. Therefore, further investigation seems to be necessary for discerning the optimal scaffold degradability to accomplish a balance between enough time for enabling initial cell nesting and complete scaffold degradation to allow its proper substitution by newly synthesized cell matrix.

Tightly related to degradability, it is also of particular importance to assess changes in the preserved scaffold matrix (if not degraded), or in the new endogenous matrix secreted by implanted cells, which replaces the scaffold. None of the reviewed studies performed an analysis of scaffold elements post-engraftment, which is hard to do as the lack of scaffold or its total integration into the host tissue makes its isolation difficult. Scaffold composition analysis should help us to understand how these changes can affect myocardial regeneration, and how variations in scaffolds can regulate and direct the mechanisms behind these, which could also explain the mechanism by which scaffolds promote cardiac recovery.

Despite the promising results obtained in most pre-clinical studies, some issues and questions remain unresolved regarding cardiac tissue engineering and the use of natural scaffolds. First, few clinical trials have tested scaffolds with or without cells [37, 38, 111, 145] (NCT00981006, NCT02139189, and NCT02057900; Table 6), which limits the ability to translate procedures from pre-clinical studies to humans. In addition, few studies have been performed in large animal MI models. These animal models could provide more realistic expectations of possible effects in humans, because their cardiovascular systems are more closely related to the human system. It is very important to determine influences on LV function, adverse remodeling, and contractility in a similar cardiovascular system. It is also crucial to investigate inflammatory responses after scaffold injection or engraftment. Because scaffold materials are typically xenogeneic or allogeneic, they might trigger inflammation and macrophage infiltration and this effect must be assessed prior to testing in humans. It is not meaningful to assess inflammatory responses in small animals because they lack human-like inflammatory responses [16]. Additionally, the absence of experimentation in large animal MI models becomes more relevant in applications where the scaffold incorporates cells, because the effects caused by cells may vary among different species. Special caution should be taken with ESCs or iPSCs because they tend to form teratomas; therefore, detailed follow-up should be conducted in large animal MI models before these treatments can be translated to clinical applications [189].

Second, long-term effects must also be evaluated to ensure maintenance of the positive effects provided by the scaffold. A study performed with synthetic scaffolds and myoblasts indicated that heart failure progression could be prevented and high numbers of cells were present after 9 months, but both effects were lost after 12 months [190]. The transitory nature of these effects, albeit using synthetic materials, should motivate new, longer studies to include several time points for evaluating scaffold properties, cell states, and cardiac function.

Finally, although several types of scaffolds and combinations of scaffolds with different cell types have been studied—and in most cases shown promising results—it remains unclear what combination of materials, cells, and growth factors or other supplements might be optimal. Ideally, a scaffold should exactly match the cardiac matrix microenvironment and three-dimensional architecture, which would favor cell attachment, proliferation, and differentiation into cardiomyogenic lineages [17]. Nevertheless, it is highly difficult to achieve this because native cardiac ECM is composed of a mixture of different proteins, glycosaminoglycans, proteoglycans, and growth factors in discrete proportions, which are dispersed in a well-organized structure with a unique three-dimensional architecture [191, 192]. Moreover, the scaffold must mechanically couple with the host myocardium and beat synchronously [17]. Decellularized myocardial ECM could probably best accomplish these conditions, but procuring it requires a decellularization process that might alter its intrinsic properties [164]. The newly emerging technology of bioprinting may facilitate the resolution of these problems. This technique employs a three-dimensional bioprinter to generate a scaffold with a predefined pattern and a well-organized, specified structure. It mixes the selected materials in the chosen proportions and provides control over many parameters, such as porosity [193, 194]. For instance, a three-dimensional bioprinter was used to create small blood vessels from a pre-determined ratio of human adult aortic smooth cells, ECs, and dermal fibroblasts. These vessels were functional and sustained mechanical integrity for up to 28 days [195]. New insights gained over the next few years about ECM structure and composition will inform improvements in bioprinting technology to enable precise selections of elements and distributions based on the native myocardial pattern. This emergent technique may provide a means to achieve the goal of recreating the myocardial ECM.

## Conclusion

Collectively, most recent advances in cardiac tissue engineering using natural scaffolds have shown promising results in myocardial regeneration after MI. These findings should facilitate the development of next-generation scaffolds with enhanced properties, either by adjusting scaffold microenvironments or by delimiting cell choices, thus enabling clinical translation of these newly developed scaffolds. Therefore, natural scaffolds are on the way to be finally implemented as a feasible and alternative MI therapy.

## Abbreviations

ATDPC: Adipose tissue-derived progenitor cells
ATDSC: Adipose tissue-derived stem cells
bFGF: Basic fibroblast growth factor
BMMNC: Bone marrow mononuclear cell
CM: Cardiomyocyte
EC: Endothelial cell
ECM: Extracellular matrix
EF: Ejection fraction
ESC: Embryonic stem cell
FS: Fractional shortening
HGF: Hepatocyte growth factor
IGF: Insulin growth factor
iPSC: Induced pluripotent stem cell
LV: Left ventricle/left ventricular
MI: Myocardial infarction
MSC: Mesenchymal stem cell
rTIMP: Recombinant tissue inhibitor of matrix metalloproteinases
Sca-1: Stem cell antigen-1
SDF: Stromal cell-derived factor
SIS: Small intestine submucosa
TGF: Transforming growth factor
UBM: Urinary bladder matrix
VEGF: Vascular endothelial growth factor
α-MHC: Alpha myosin heavy chain
